# Effect of *SCN1A*and *SCN2A* gene polymorphisms on the efficacy of valproic acid treatment in Chinese children with epilepsy

**DOI:** 10.1371/journal.pone.0304869

**Published:** 2024-06-05

**Authors:** Zejun Bao, Huanzhou Li, Jing Hu, Ru Zhao, Ling Yan, Aibin Zheng

**Affiliations:** Changzhou Children’s Hospital Affiliated to Nantong University, Changzhou, Jiangsu, China; University of South Australia, AUSTRALIA

## Abstract

**Objective:**

Epilepsy patients exhibit considerable differences in their response to sodium valproate (VPA) therapy, a phenomenon that might be attributed to individual genetic variances. The role of genetic variations, specifically in sodium channels encoded by SCN1A and SCN2A genes, in influencing the effectiveness of VPA in treating epilepsy is still debated. This research focuses on examining the impact of these genetic polymorphisms on the efficacy of VPA therapy among pediatric epilepsy patients in China.

**Methods:**

Five single nucleotide polymorphisms (SNPs), including SCN1A (rs10188577, rs2298771, rs3812718) and SCN2A (rs2304016, rs17183814), were genotyped in 233 epilepsy patients undergoing VPA therapy. The associations between genotypes and the antiepileptic effects of VPA were assessed, with 128 patients categorized as VPA responders and 105 as VPA non-responders.

**Results:**

In the context of VPA monotherapy, SCN1A rs2298771 and SCN2A rs17183814 were found to be significantly associated with VPA response (*P*< 0.05).

**Conclusion:**

Our study suggests the findings of this investigation indicate that the polymorphisms SCN1A rs2298771 and SCN2A rs17183814 could potentially act as predictive biomarkers for the responsiveness to VPA among Chinese epilepsy patients.

## 1. Introduction

Epilepsy is a pervasive neurological condition impacting around 65 million individuals worldwide. This disorder places a heavy burden on those affected, encompassing disability from seizures, increased mortality risk, comorbid health issues, societal stigma, and significant financial costs. The primary strategy for managing epilepsy involves the use of antiepileptic drugs (ASMs). Nonetheless, it is noteworthy that despite receiving treatment, about 30% of patients still suffer from seizures [1]. Resistance to ASMs is a complex phenomenon, with growing evidence suggesting that genetic variations significantly impact the efficacy of drug therapy, affecting both pharmacokinetic and pharmacodynamic processes [2]. Among the various treatments, valproic acid (VPA) is often the drug of choice for controlling different types of seizures, including both generalized and focal seizures. However, the effectiveness of VPA varies significantly among patients, with differences in blood levels of VPA only partially explaining these pharmacodynamic variations.

Epilepsy is intrinsically related to abnormalities in ion channel functions, with Nav channels being primary targets for several frontline ASMs including carbamazepine, oxcarbazepine, phenytoin, lamotrigine, and VPA. Nav channels are composed of α and β subunits, with the α subunit playing a pivotal role in their functionality. VPA’s therapeutic action is attributed to its interaction with the α subunit [3]. Importantly, the central nervous system harbors two principal Nav channel isoforms, Nav1.1 and Nav1.2, encoded by SCN1A and SCN2A genes [9], respectively. The SCN1A gene, associated with the α-subunit of Nav1.1 channels, has a strong correlation with epilepsy. Variations in SCN1A can result in a spectrum of hereditary seizure disorders. Similarly, variations in the SCN2A gene, responsible for the α-subunit of Nav1.2 channels, are linked to a range of epilepsy syndromes [4]. These genetic variations in SCN1A and SCN2A are thought to underlie the individual differences in response to AED treatment.

This investigation aims to delve into the influence of SCN1A and SCN2A gene polymorphisms on the effectiveness of VPA in managing epilepsy among the Han Chinese population. By identifying genetic markers predictive of VPA resistance, this study seeks to pave the way for tailored VPA therapy, enhancing treatment outcomes for patients with epilepsy.

## 2. Materials and methods

### 2.1. Patient inclusion and exclusion criteria

The study incorporated 233 pediatric patients of Chinese Han ethnicity diagnosed with epilepsy, recruited from our neurology department. Eligibility for participation required a documented medical history of at least two clinically verified spontaneous seizures. These children were initially treated exclusively with VPA monotherapy. Of them, 105 were subsequently administered at least three distinct antiepileptic drugs (ASMs) following an insufficient response to VPA. At the initial consultation, detailed demographic and clinical profiles were compiled, encompassing data on gender, age, body weight, height, seizure categorizations, the onset age of seizures, familial epilepsy history, comprehensive medical and neurological backgrounds, along with seizure frequency and duration. Treatment plans for epilepsy were also documented. Follow-up assessments were scheduled quarterly over a year of VPA treatment, recording the ASMs dosage at each visit, adherence to the prescribed medication regimen, and the treatment’s effectiveness. Blood samples for measuring VPA concentrations were collected at the lowest concentration point when the maximum maintenance dose was reached during the study.Criteria for exclusion included individuals with known adverse reactions to VPA or those with a medical history indicative of progressive neurological disorders. Participants with a record of poor medication adherence were likewise omitted. Enrollment in the study was predicated on acquiring written informed consent from either the patients themselves or their legal guardians. This study was approved by the ethics Committee of Changzhou Children’s Hospital affiliated to Nantong University.

### 2.2 Definition of resistance and reactivity

Clinical efficacy evaluation [4]: ① Response: With a fixed dose of VPA treatment for 1 year, achieving stable blood drug concentrations and reducing seizure frequency by more than 50% within this period. Drug resistance: Despite taking the maximum dose of VPA for 1 year, the condition remains uncontrolled with seizure frequency exceeding 50%, necessitating the use of alternative anti-epileptic drugs.

### 2.3 Genotyping

#### 2.3.1 Detection of VPA blood concentration

The blood concentration of VPA was measured using LC-MS chromatography.

The following day, 2 ml of fasting venous blood was collected from the patient prior to medication intake. After centrifugation to obtain plasma, it was transferred to an EP tube. To this, 20 μL of VPA standard solution and, subsequently, 50 μL of IS solution were added with precise mixing. After adding 600 μL of acetonitrile solution and vortex mixing uniformly, the supernatant was obtained through centrifugation. The VPA blood concentration was then determined by LC-MS.

#### 2.3.2 Detection of gene polymorphism

3 ml of peripheral venous blood was utilized for DNA extraction using a whole blood genomic DNA extraction kit (Thermo Fisher Technology Co., LTD.). Primers were designed for SNPs including SCN1A (rs10188577, rs2298771, rs3812718), SCN2A (rs2304016, rs17183814). The PCR amplification protocol involved an initial denaturation at 95°C for 3 minutes, followed by 35 cycles of denaturation at 94°C for 30 seconds, annealing for 30 seconds, and extension at 72°C for 30 seconds, with a final extension at 72°C for 10 minutes. PCR products were analyzed using 1.5% agarose gel electrophoresis and sequenced with a 3730XL automatic DNA sequencer (ABI, USA).

### 2.4 Statistical analysis

SPSS 16.0 software package was used for data analysis and processing. Count data and distribution of analyzed genotypes were tested by *χ*^*2*^ test. Measurement data were expressed as mean±standard error (Mean±SEM). For normally distributed parameters, two-sample t-test was used for comparison between two groups, and one-way ANOVA was used for comparison between multiple groups; non-parametric tests were used for skewed distribution variables. Comparison of parameters between the two groups before and after treatment was performed using paired t-test. p<0.05 was considered statistically significant.

## 3. Results

### 3.1 Comparison of baseline data between VPA monotherapy responder and resistant groups

Due to incomplete follow-up and indeterminate drug responses, a total of 84 patients were excluded, resulting in 233 patients being ultimately included in the study. Except for the blood concentration of valproic acid, no significant statistical difference was observed in the general data between the two groups (*P* > 0.05). See Table 1.

**Table 1:** Demographic and clinical characteristics of patients with epilepsy treated with VPA.

hallmark	VPA Response Group (*n* = 128)	VPA-resistant group (*n* = 105)	*P*-value
Male, n (%)	81 (64.8)	78 (60.9)	0.525
Age (years)	8.23±3.02	8.33±3.24	0.053
Type of seizure (primary/secondary)	70/58	66/39	0.208
Epilepsy duration (years)	3.63 ±0.80	3.61±0.73	0.349
VPA Duration of treatment (years)	1.44± 0.28	1.44±0.28	0.789
Daily dose of VPA (mg/kg)	21.74±4.85	18.08±9.36	0.630
VPA trough concentration(μg/mL)	68.61±11.66	57.88±9.56	0.016

### 3.2 Comparison of allele and genotype frequencies of SCN1A in the VPA-responder and VPA-resistant groups

**Fig 1:**
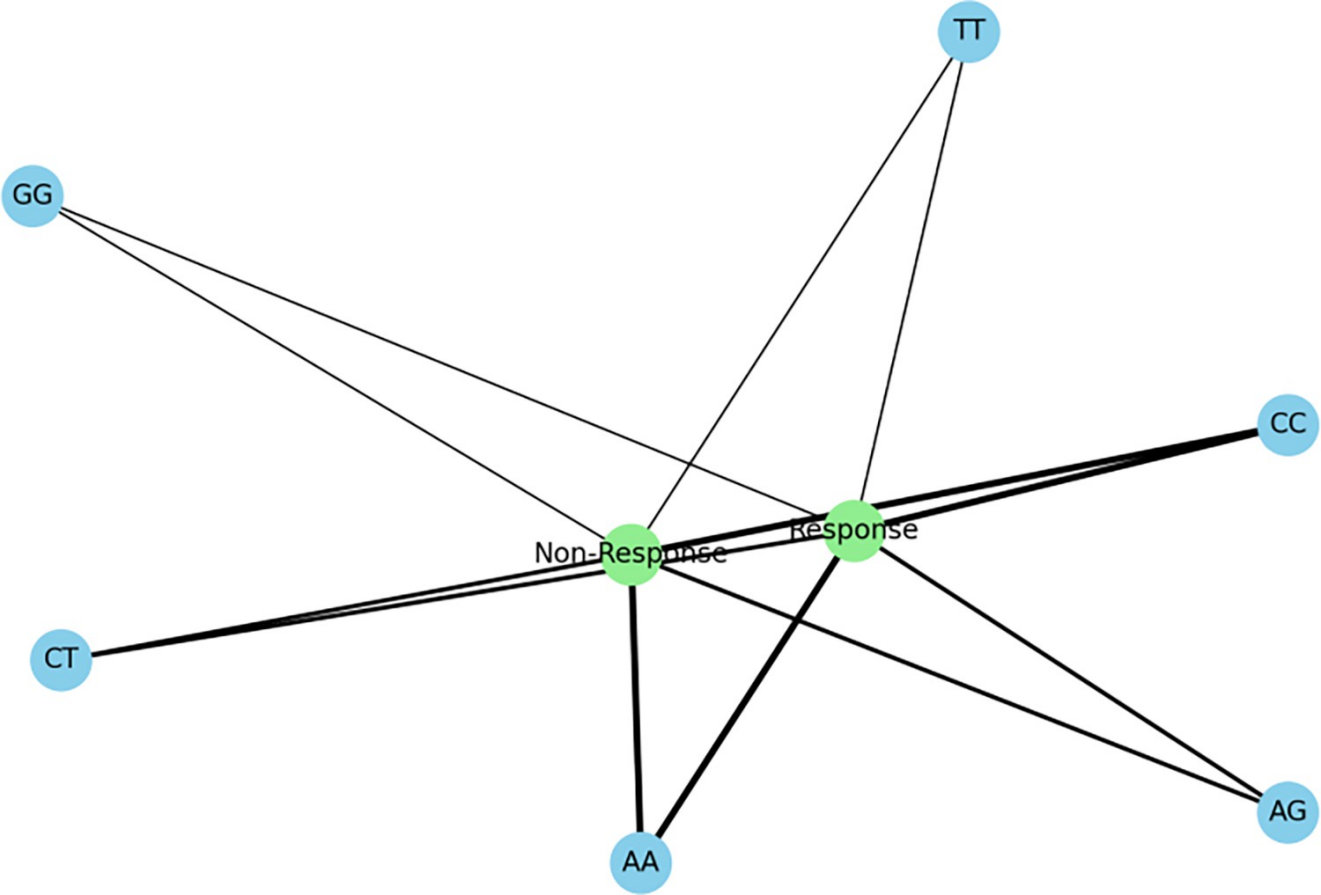
Association of SCN1A genotypes with clinical outcomes.

**Fig 2:**
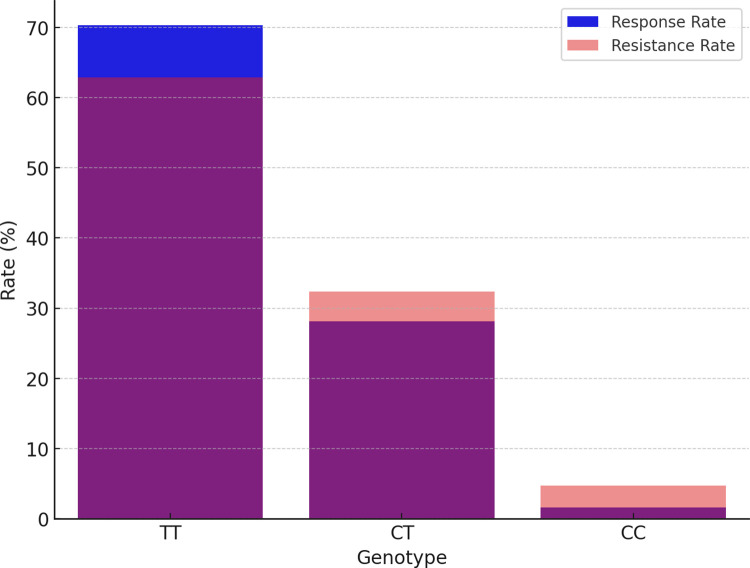
SCN1A rs2298771 T > C treatment response rate comparison.

**Fig 3:**
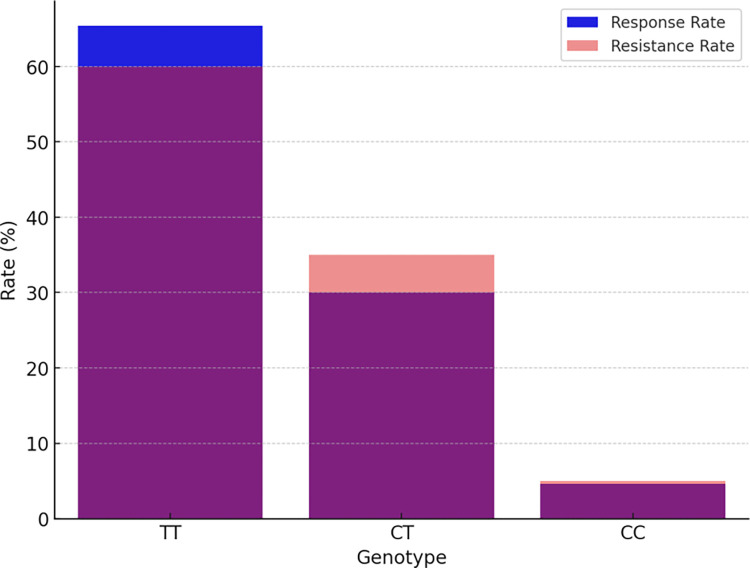
SCN1A rs10188577 T > C treatment response rate comparison.

**Fig 4:**
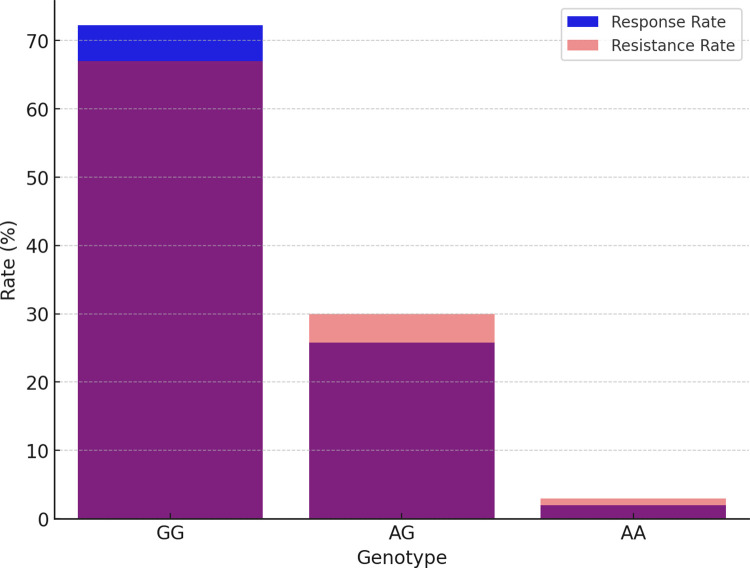
SCN1A rs3812718 G > A treatment response rate comparison.

In our follow-up analysis of the SCN1A gene polymorphisms, we particularly focused on three loci: rs10188577, rs2298771, and rs3812718. The association between these loci and clinical efficacy is depicted in Table 2 and Fig 1. The distribution of alleles and genotypes showed similarity between the valproic acid (VPA) responsive group and the VPA resistant group. However, the analysis revealed a significant association between SCN1A rs2298771 and a positive response to monotherapy with VPA. Specifically, when examining the genotype frequency of the rs2298771 T > C polymorphism, carriers of the TT genotype were found to be more prevalent in the VPA responsive group (*P* = 0.043). Conversely, in the dominant model, the CT and CC genotypes were more common in the VPA resistant group compared to the VPA responsive group (*P* = 0.047), as shown in Fig 2. Furthermore, we conducted further analyses on the polymorphisms of other relevant gene loci, and the relationships between rs10188577 T > C、 rs3812718 G > A loci and the response to VPA treatment are detailed in Figs 3 and 4. These charts not only display the impact of individual gene loci but also provide us with a holistic view, aiding in understanding the potential impact of multiple gene interactions on the effectiveness of VPA treatment. Through these detailed analyses and charts, we emphasize the potential importance of genotype in predicting the response of children to VPA treatment. As shown in Figs 1 to 4, these findings offer valuable insights into how genetic polymorphisms influence epilepsy treatment and may guide future personalized treatment strategies.

**Table 2:** Distribution of *SCN1A* genotypes and alleles in the VPA-responder and VPA-resistant groups of patients receiving VPA monotherapy.

genetics	single nucleotide polymorphism	Genotype/allele	VPA response *n*(%)	VPA resistance *n*(%)	*P*-value
SCN1A	rs10188577 T > C				
		TT	90 (70.3)	66 (62.9)	0.248
		CT	36 (28.1)	34 (32.4)	
		CC	2 (1.6)	5 (4.8)	
	phanero-	CT + CC	38 (29.7)	39 (37.1)	0.263
		T	216 (84.4)	166 (79)	0.147
		C	40 (15.6)	44 (21)	
	rs2298771 T > C				
		TT	109 (85.2)	78 (74.3)	0.043
		CT	16 (12.5)	26 (24.8)	
		CC	3 (2.3)	1 (0.9)	
	phanero-	CT + CC	19 (14.8)	27 (25.7)	0.047
		T	234 (91.4)	182 (86.7)	0.132
		C	22 (8.3)	28 (13.3)	
	rs3812718 G > A				
		GG	25 (19.5)	19 (18.1)	0.448
		GA	63 (49.2)	60 (52.8)	
		AA	40 (31.2)	26 (28.3)	
	phanero-	AA + GA	103 (80.5)	86 (81.9)	0.867
		G	113 (44.1)	98 (46.7)	0.640
		A	143 (55.9)	112 (54.7)	

### 3.3 Comparison of allele and genotype frequencies of SCN2A in the VPA-responder and VPA-resistant groups

**Fig 5:**
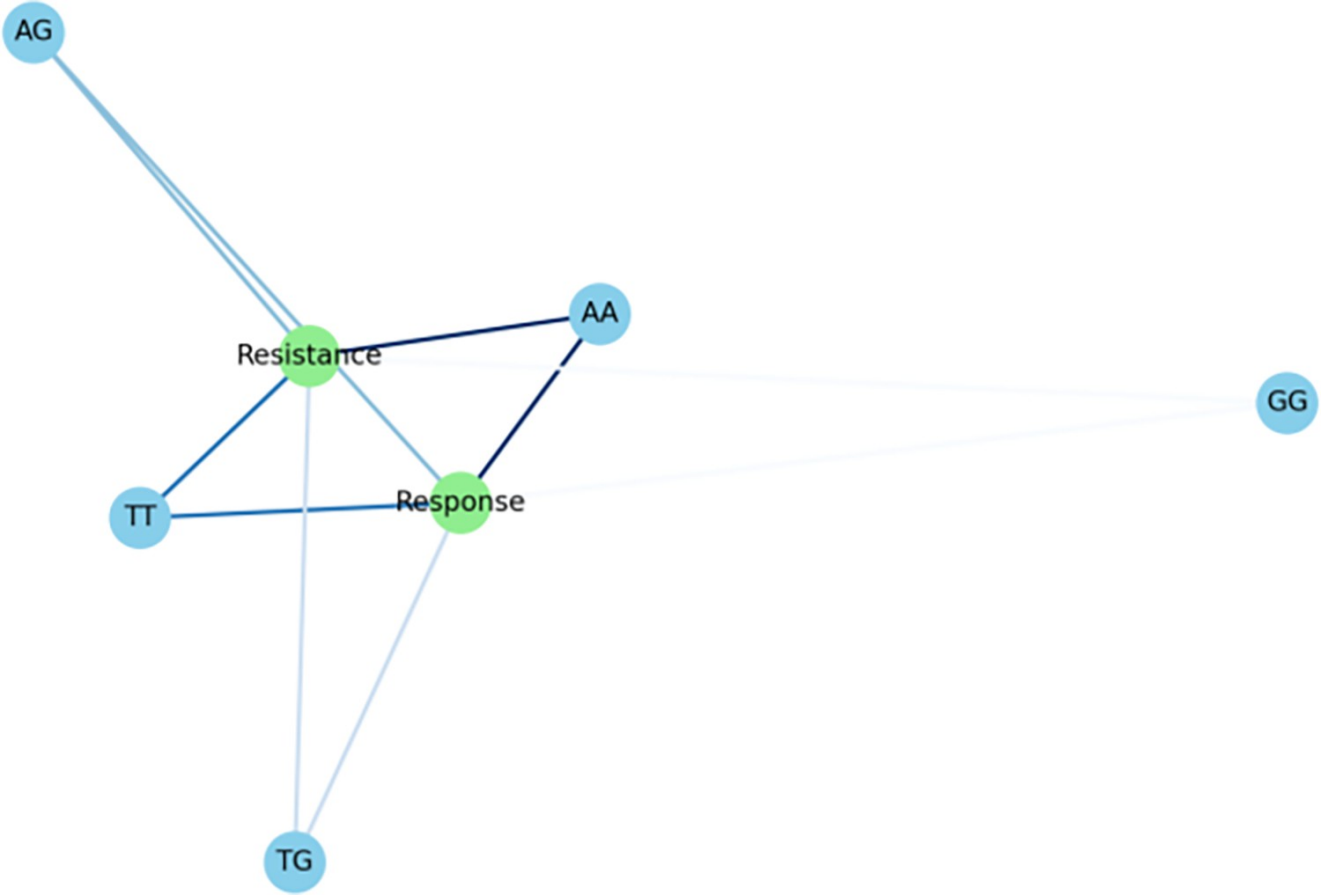
Association of SCN2A genotypes with clinical outcomes.

**Fig 6:**
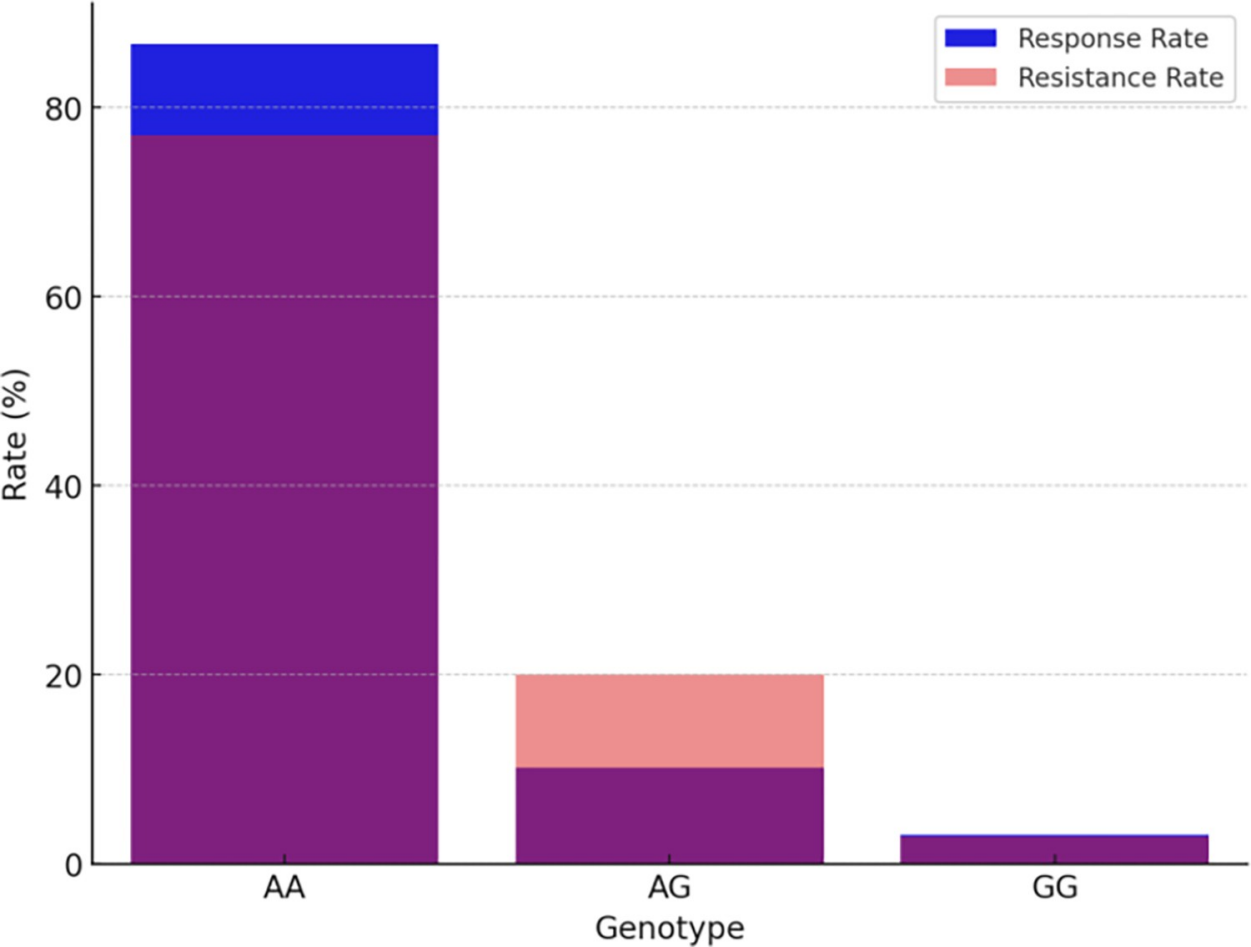
SCN2Ars2304016 A > G treatment response rate comparison.

**Fig 7:**
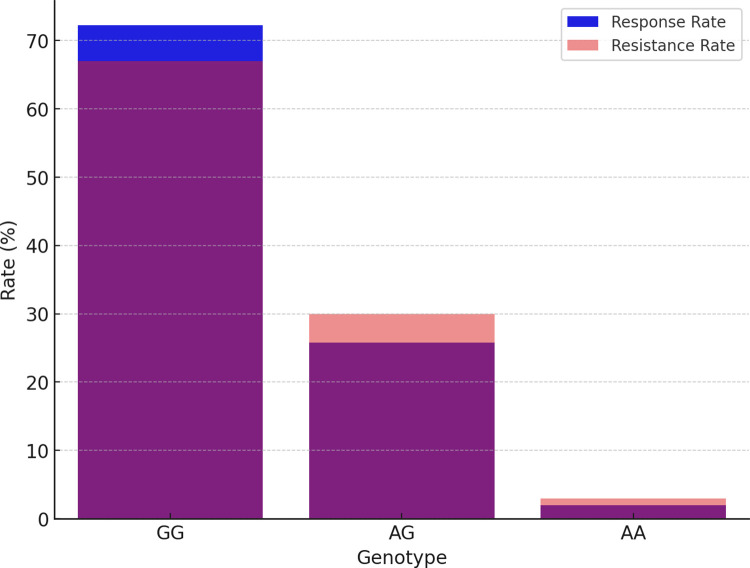
SCN2A17183814 G > A treatment response rate comparison.

In our subsequent analysis of the SCN2A gene polymorphism, we specifically focused on the rs2304016 and rs17183814 loci; the correlation between these two loci and clinical efficacy is depicted in Table 3 and Fig 5. Our data reveal that there is no significant difference in the allele and genotype distribution at the SCN2A rs2304016 locus between the valproic acid (VPA) responsive and resistant groups, suggesting that mutations at this locus might not decisively influence children’s response to VPA treatment, as illustrated in Fig 6. However, the situation at the SCN2A rs17183814 locus differs. We observed a significantly higher proportion of individuals with the GG genotype in the VPA responsive group compared to the VPA resistant group (*P* = 0.025), as depicted in Fig 7. This finding indicates that the GG genotype may be associated with a positive response to VPA treatment. Furthermore, employing the dominant genetic model analysis revealed that the AA+AG genotype was more prevalent in the VPA resistant group than in the responsive group (*P* = 0.044), further underscoring the potential significance of the rs17183814 locus in predicting VPA treatment outcomes. Through the detailed analysis presented in Figs 5 to 7, we not only understand how a single gene locus influences the response to VPA treatment but also gain insight into the potential comprehensive impact of multi-gene interactions on treatment outcomes. These analyses highlight the importance of considering specific genotypes in formulating future personalized treatment strategies.

**Table 3:** Distribution of *SCN2A* genotypes and alleles in the VPA-responder and VPA-resistant groups of patients receiving VPA monotherapy.

genetics	single nucleotide polymorphism	Genotype/allele	VPA response n(%)	VPA resistance n(%)	*P*-value
SCN2A	rs2304016 A > G				
		AA	111 (86.7)	81 (77.1)	0.106
		AG	13 (10.2)	21 (20.0)	
		GG	4 (3.1)	3 (2.9)	
	phanero-	AG + GG	17 (13.3)	24 (22.9)	0.060
		A	235 (91.8)	183 (87.1)	0.125
		G	21 (6.2)	27 (12.9)	
	rs17183814 G > A				
		GG	110 (85.9)	79 (75.2)	0.025
		AG	12 (9.4)	23 (21.9)	
		AA	6 (4.7)	3 (2.9)	
	phanero-	AG + AA	18 (14.1)	26 (24.8)	0.044
		G	232 (90.6)	181 (86.2)	0.144
		A	24 (9.4)	29 (13.8)	

## 4. Discussion

Epilepsy is recognized as an ion channel disorder, and voltage-gated ion channels constitute common targets for the action of antiepileptic drugs (ASMs). Variants within the SCN1A and SCN2A genes have been proposed as potential contributors to antiepileptic drug resistance [5]. Notably, a missense mutation in the SCN1A exonic region (SCN1A rs2298771) results in the substitution of threonine with alanine through a G to A base change [6]. Similarly, an exonic region mutation in SCN2A (SCN2A rs17183814) leads to the conversion of arginine to lysine by altering the G base [7]. These two single nucleotide polymorphisms (SNPs) have the potential to impact the structural and functional characteristics of Nav channels, thereby influencing the therapeutic effectiveness of ASMs [8]. In addition to these variants, SCN1A rs10188577, rs3812718, and SCN2A rs2304016 are common SNPs within the intronic region that play significant roles in the expression and regulation of SCN1A and SCN2A genes [9]. The relationship between polymorphisms within SCN1A and SCN2A genes and drug resistance has been extensively studied, but the findings remain inconclusive. While numerous studies have explored the correlation between these polymorphisms and ASMs response, including VPA, few have specifically focused on the response to VPA, and the results have been conflicting.For instance, a cohort study conducted in Hong Kong, China, and Malaysia involving patients treated with VPA monotherapy did not reveal a significant impact of SCN1A gene polymorphisms on VPA response [10]. In contrast, a study in China found that the rs3812718 locus of the SCN1A gene was the only SNP associated with AED response, including VPA [11]. Furthermore, another Chinese epilepsy cohort study demonstrated associations between SCN1A rs3812718 and SCN2A rs2304016 with VPA response [12].

Valproic acid (VPA) is a broad-spectrum antiepileptic drug widely employed in the management of various seizure types. Individuals, however, exhibit substantial variability in their response to VPA, with pharmacogenetics playing a pivotal role in explaining this variation. Consistent with recent research [13], the TT genotype of rs2298771 may serve as a protective factor in the treatment of childhood epilepsy with VPA. Our study found a significant association between SCN1A rs2298771 T > C and drug response in both VPA monotherapy and VPA polypharmacy. Patients carrying the rs2298771 TT genotype displayed heightened sensitivity to VPA treatment, resulting in improved seizure control compared to CT and CC genotype carriers.

Given the crucial role of Nav1.2 in epileptogenesis, we also sought to establish a potential correlation between SCN2A gene polymorphisms and VPA drug resistance within our cohort. Our findings suggest that SCN2A rs17183814 may act as a risk factor for VPA monotherapy resistance, with GG genotype carriers displaying an increased likelihood of resistance to VPA therapy. This study underscores the influence of SCN2A rs17183814 on VPA treatment efficacy.Effective antiepileptic treatment necessitates the maintenance of adequate drug concentrations, emphasizing the importance of therapeutic drug monitoring in epilepsy therapy. In our study population, VPA blood concentrations in patients within the responder group did not significantly differ from those in the resistant group. Drugs exert their pharmacological effects by binding to specific targets, and alterations in these targets can modify drug actions. Given the diversity of cellular targets through which VPA operates, encompassing channels, receptors, and pathways involved in gene expression regulation, further research is imperative to elucidate the precise mechanisms underpinning its antiepileptic actions.

In conclusion, our study has highlighted the association between the SCN1A rs2298771 polymorphism and VPA monotherapy response, with the TT genotype serving as a protective factor for improved antiepileptic response. Additionally, the SCN2A rs17183814 polymorphism appears to influence VPA efficacy, with the GG genotype representing a potential risk factor for VPA treatment resistance. Pharmacogenomic diagnosis stands as a crucial component of precision medicine. However, due to the relatively modest sample size in our study, the confirmation of these associations warrants prospective trials with larger populations. Identifying patients with epilepsy who would derive the greatest benefit from VPA therapy may involve the consideration of SCN1A rs3812718 and SCN2A rs2304016 genotypes.

## Supporting information

S1 Dataset (XLSX)
